# Within-Plant Distribution of Two-Spotted Spider Mites, *Tetranychus urticae* Koch (Acari: Tetranychidae), on Strawberries: Decision of an Optimal Sampling Unit

**DOI:** 10.3390/insects13010055

**Published:** 2022-01-04

**Authors:** Yong-Seok Choi, Min-Jung Kim, Sunghoon Baek

**Affiliations:** 1 Bioenvironmental Division, Chungnam Agricultural Research and Extension Services, Yesan 32418, Korea; yschoi92@korea.kr (Y.-S.C.); 2017-24294@snu.ac.kr (M.-J.K.); 2 Department of Plant Medicals, Andong National University, Andong 36729, Korea; 3 Department of Industrial Entomology, Korea National College of Agriculture and Fisheries, Jeonju 54874, Korea

**Keywords:** with-plant distribution, *Tetranychus urticae*, strawberry, sampling unit

## Abstract

**Simple Summary:**

Although two-spotted spider mite (*Tetranychus urticae*) is a major pest for strawberry, a popular fruit, there is limited information about the sampling unit for *T. urticae* in strawberries. Thus, the objective of this study was to suggest an optimal sampling unit of *T. urticae* in strawberries based on characteristics of their within-plant distribution. The sampling unit was changed according to developmental stage of *T. urticae*: the sixth oldest trifoliate leaf (eggs), the fifth oldest leaf (immatures and adults), and the fifth or sixth oldest leaf (all stages). For management purposes, the required sample numbers within a strawberry were one leaflet and two leaflets for eggs and motiles, respectively. Any leaflets in a trifoliate leaf could be selected among three leaflets of the selected sampling unit. However, the sample number for the research purpose should be determined by counting the number of *T. urticae* on a few leaflets of the suggested sampling unit. By using the optimal sampling unit for *T. urticae* and its required sample number, its sampling could be more precise to estimate *T. urticae* density in strawberries. Moreover, this precise sampling could improve the efficiency of *T. urticae* management.

**Abstract:**

It is known that two-spotted spider mite (*Tetranychus urticae*), a major pest of strawberry, is difficult to manage. This study was conducted to determine the optimal sampling unit to increase management efficiency of *T. urticae* in strawberries. The sampling unit was determined by characterizing within-strawberry distribution of *T. urticae* and by comparing coefficient of variation (CV) and correlation coefficient (*r*^2^) among potential sampling units. There was a significant (*p <* 0.05) difference in densities within a strawberry according to the leaf age. However, there was no significant difference in its density within a trifoliate leaf. More *T. urticae* were found on young-fully-opened (moderately old) leaves than on young and old ones. Moreover, these leaves had lower CV and *r*^2^ values than others. More specifically, optimal sampling units of *T. urticae* were fifth, fifth or sixth, and sixth oldest leaves for motiles (immatures and adults), all stages (motiles and eggs), and eggs, respectively. The required sampling number should be determined depending on the density. However, for management purposes, one and two leaflets would be acceptable for eggs and motiles, respectively. By using this small number of suitable sampling unit, the sampling and management for *T. urticae* in strawberries could be more efficient than before.

## 1. Introduction

Strawberry (*Fragaria* × *ananassa* Duchesnae) is hybrid crop of the genus *Fragaria* in the order Rosales that grows worldwide [1]. Although mites, aphids, moths, thrips, whiteflies, and leafminers are known as major pests in strawberries, farmers consider the two-spotted spider mite, *Tetranychus urticae* Koch, as the worst pest for strawberries among all arthropod pests in Korea [2]. There are multiple reasons for the notorious reputation of *T. urticae* in Korea. One of the most important reasons is serious resistance of *T. urticae* to acaricides in Korea [3,4]. In addition, its small size, ability to rapidly increase its population, short generation time, and large population variation within and between farms can make *T. urticae* a difficult pest to control in diverse crops [5,6].

The two-spotted spider mite is a polyphagous and herbivorous pest that can cause serious economic damages to diverse plants [7]. Multiple trials have been conducted to estimate its populations on cucumbers [8], roses [5], ivy geraniums [9], peppermint [10], apples and pears [6], and strawberries [11,12]. Accurately estimating *T. urticae* populations is an important and basic step in its management strategy [13]. However, a more fundamental step is to decide a reasonable and efficient sampling unit on the target plant before estimating its population in any pest management programs [14]. This sampling unit affects characteristics of a population, components of sampling programs (e.g., sampling techniques, sampling number, spatial distribution of a target pest, and sampling timing), and management plans [14,15,16,17]. The sampling unit is generally determined based on within-plant distribution, although a few other factors such as its size, sampling consistency, efficiency, and precision level, sampling stability, and possibility to delineate contaminated areas should also be considered [14,18].

Within-plant distribution of *T. urticae* has been analyzed in three studies [5,9,10]. Two studies [5,10] have concluded that its within-plant distribution is not significantly different vertically. So [5] has mentioned that more individuals of *T. urticae* are found on lower leaves than on upper ones in its low density. However, Opit et al. [9] have shown that more mites are present on young-fully-opened leaves than on young or old leaves, indicating the presence of a vertical preference of *T. urticae* within a plant. Based on results of within-plant distribution, Opit et al. [9] have suggested an optimal sampling unit of *T. urticae* in ivy geranium as any young-fully-opened lead within a plant. Choe et al. [12] have suggested that any one leaflet is an optimal sampling unit on strawberries by simply comparing relative net precision among one, two, and three leaflets within a trifoliate leaf without analyzing within-plant distribution. Although within-plant distribution and sampling unit of *T. urticae* were studied, there was no consistent within-plant distribution characteristic or sampling unit for *T. urticae*. Moreover, no study has suggested the minimum number to satisfy the desired precision level within a plant. Thus, this study was conducted to find out characteristics of within-plant distribution and to suggest an optimal sampling unit of *T. urticae* in strawberries.

## 2. Materials and Methods

### 2.1. Study Sites

Two greenhouses in this study were located at Noseoung-Myeon, Nonsan-Si, Chungcheonnam-Do, Korea. Both farm owners grew strawberries on beds of approximately one meter off the ground with aquiculture. The variety of strawberries in both farms was “Kingsberry” which was newly developed at Chungnam Agricultural Research and Extension Services. The main characteristic of this variety is a huge fruit size which is similar to the size of a large chicken egg. The farm owners transplanted this variety seedlings on the same day, 25 August 2020. Except for acaricide, they managed their farms with their own ways, including composition of nutrient solution, management timing, pesticides, and so on. The size of one greenhouse (first farm) was about 800 m^2^ (10 m by 80 m) with seven rows. The other (second farm) was approximately 720 m^2^ (9 m by 80 m) with six rows. In each row, two strawberries were planted every 10 cm. Harvesting began in early December.

### 2.2. Sampling

In both greenhouses, the sampling timing was determined by farm owners. They allowed one week just before the first acaricide application. The sampling number was also suggested by farm owners. For the first farm, a total of 32 plants were randomly selected every 10 m and observed. Among seven rows, the outer four rows were used. The sampling date was 18 January 2021. For the second farm, 33 plants were randomly observed in the same way as for the first farm. In this second farm, the outer four rows were used among six rows. Sampling date was 19 January 2021.

**Figure 1:**
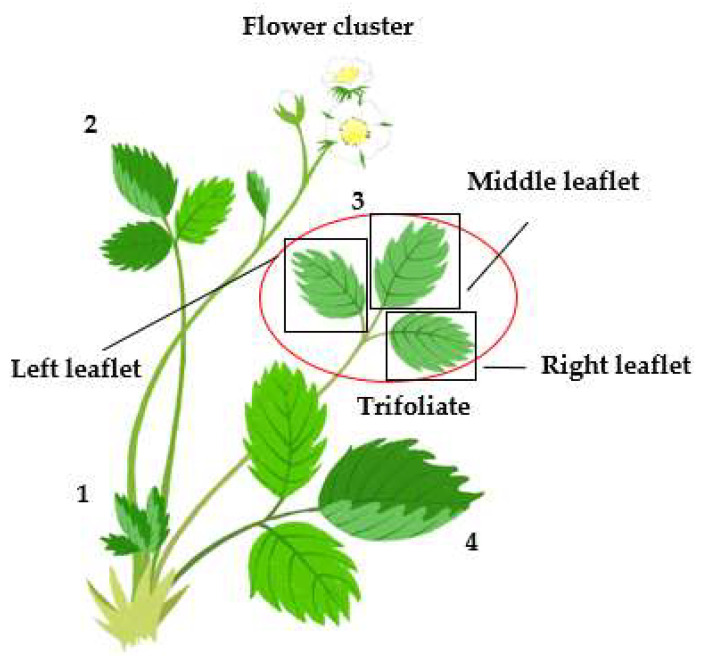
Diagram of the trifoliate leaf and its relative positions within a strawberry plant for sampling *T. urticae* in this study. Within a trifoliate leaf, the left and right leaflets were separated based on the middle leaflet. The numbers above trifoliate leaves indicate the numbering for sampling *T. urticae*. Numbering was started from newly developed leaves to older ones according to the leaf age. The flower cluster was separately recorded.

From the selected plants, all leaves and flower clusters were collected individually. To collect all habitable units of *T. urticae*, a numbering system was used (Figure 1). Generally, a new stem is developed in the hub within a strawberry plant and then erected. Each stem has a leaflet cluster (trifoliate) composed of three leaflets (Figure 1). As time goes by, the stem with old leaves will droop. Thus, numbering was started from newly developed stems in the middle to older ones within a strawberry (Figure 1). Within a trifoliate leaf, the left and right leaflets were separated based on the middle leaflet (Figure 1). Each leaflet and flower cluster were carefully cut with hand pruners (P-300, Hwashin Metal IND Co.; Daegu, Korea) and collected individually using zipper bags (Ziploc, 16.5 × 14.9 cm, Thai Griptech Co., Ltd., Bangkok, Thia). These zipper bags were preserved in a refrigerator in the laboratory of Bioenvironmental Division, Chungnam Agricultural Research and Extension Services before counting *T. urticae* to prevent population changes. Mites were counted under an optical microscope (Leica S8 APO, Leica Microsystems; Wetzlar, Germany) and recorded for eggs and motiles. Motiles included adults and immatures because it was difficult to distinguish these developmental stages exactly.

### 2.3. Within-Plant Distribution

As a step of preliminary data analysis, densities of *T. urticae* eggs, motiles, and totals of eggs and motiles were compared between strawberries from the two greenhouses with ANOVA [19]. There was no statistically significant difference in egg density (*t* = 0.746, df = 63, *p* = 0.229) or total density (*t* = 1.411, df = 63, *p* = 0.082) between the two farms. However, there was a statistically significant difference in motile density (*t* = 2.257, df = 63, *p* = 0.014) between the two farms. Thus, an analysis was conducted for its egg and total (egg + motile) densities by combining the two farms’ data whereas data for its motiles were analyzed separately for each farm. Differences in *T. urticae* densities according to leaf age and leaflet location were subjected to ANOVA [19]. The leaflet size was not considered in these analyses.

### 2.4. Sampling Unit

To suggest an optimal sampling unit, two factors were considered with the density of *T. urticae*. The first factor was coefficient of variation (CV, standard deviation/mean) of *T. urticae* density according to the leaf age of strawberries. The sampling unit with a lower CV value has two ecological meanings—i.e., a relatively higher possibility to find *T. urticae* and/or a smaller sample variance than the one with a higher CV value in sampling [14,20]. The second factor was coefficient of determination (*r*^2^) estimated from the linear relationship between *T. urticae* densities on a selected trifoliate leaf and whole leaves within a plant. The sampling unit with a higher coefficient determination value has increased representativeness compared with the one with a lower coefficient determination [14].

### 2.5. Required Sample Number

The required sample number is the smallest number of sampling units to achieve desired precision level [15]. Although multiple equations were available, the following equation was used in this study because it was difficult to define the aggregation degree of *T. urticae* within a strawberry. The equation [21] was

*N* = 1/(*D*^2^ × *m*), (1)

In this equation, *N* = required sample number, *D* = precision level (mean/standard error), and *m* = mean density of *T. urticae*.

## 3. Results

### 3.1. Characteristics of Within-Plant Distribution

More *T. urticae* were found on moderately old (young-fully-opened) leaflets than on young or old ones: eggs (Table 1, *F* = 5.37; df = 11, 578; *p <* 0.0001); motiles in the first farm (Table 2, *F* = 3.30; df = 10, 281; *p* = 0.0005); motiles in the second farm (Table 3, *F* = 1.99; df = 11, 285; *p* = 0.0296); both eggs and motiles (Table 4, *F* = 5.30; df = 11, 578; *p <* 0.0001). However, there was no significant difference in *T. urticae* density according to the relative location of leaflets among trifoliate leaflets (Table 5): eggs (*F* = 0.60; df = 2, 192; *p* = 0.5494); motiles in the first farm (*F* = 0.43; df = 2, 93; *p* = 0.6548); motiles in the second farm (*F* = 0.62; df = 2, 96; *p* = 0.5415); both eggs and motiles (*F* = 0.68; df = 2, 192; *p* = 0.5059).

### 3.2. Optimal Sampling Unit

For *T. urticae* at egg stage, the sixth oldest trifoliate leaf had the lowest CV but the highest *r*^2^ value in case of enough replication (i.e., 50), indicating that this leaf was the best sampling unit within a strawberry plant (Table 1). However, its motiles showed different results. In the first farm, the CV value was the lowest on the fifth oldest leaf but the *r*^2^ value was the highest on the fourth oldest one in the case of more than 25 replications (Table 2). In the second farm, the CV value was the lowest on the third oldest leaf. However, the *r*^2^ value was the highest for the fourth oldest leaf in the case of more than 25 replications (Table 3). In all developmental stages, the CV value was the lowest on the fifth oldest leaf while the *r*^2^ value was the highest on the second oldest leaf within a plant in case of more than 50 replications (Table 4).

### 3.3. Required Sample Number

**Figure 2:**
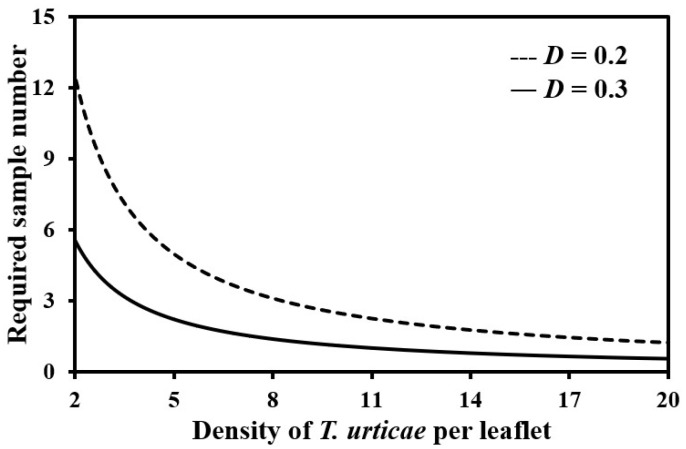
Required sample number according to densities of *T. urticae* per leaflet at fixed precision levels (*D*) of 0.2 (research purposes) and 0.3 (management purposes).

The required sample number changed according to densities of *T. urticae* (Figure 2). In this study, the average egg densities of *T. urticae* per leaflet at the selected sampling unit were roughly 22.0 and 17.0 at the first and second farms, respectively. Thus, the sample numbers for *T. urticae* egg stage were 0.5 and 0.7 leaflets for the first and second farms of this study, respectively, for management purposes (i.e., *D* = 0.3 [15]). For research purposes (i.e., *D* = 0.2 [15]), the required number increased to be 1.1 and 1.5 leaflets for the first and second farms, respectively. For the motile stage, minimum numbers were 0.7 and 1.8 leaflets to meet desired precision level of management and the number were 1.5 and 4.0 leaflets to meet the level of research in the first and second farms, respectively. The reason was that the motile densities were 16.9 and 6.2 at the first and second farms, respectively. For all stages, the required sample number was less than one regardless of farms or purposes because the densities were 38.9 and 23.2 at the first and second farms, respectively.

## 4. Discussion

This study showed within-plant distributions of *T. urticae* by observing all habitable areas of a strawberry plant. According to leaf age, there was a significant (*p <* 0.05) difference in the density of *T. urticae.* However, there was no significant (*p >* 0.05) difference in *T. urticae* density among three leaflets within a trifoliate leaf. These results indicate that the sampling unit of *T. urticae* should be determined by the leaf age and that it might not be affected by relative locations of similar aged leaflets.

The characteristics of within-plant distribution of *T. urticae* on multiple hosts have been studied before. So [5] has reported that *T. urticae* is more abundant in the lower third and absent from the upper third of roses in conditions of low *T. urticae* density. That study [5] also showed that such a trend disappeared as density increased. Opit et al. [9] have insisted that there are more *T. urticae* on young-fully-opened leaves than on young or old leaves of ivy geranium plants. However, Tollerup et al. [10] have found no statistical difference (*p >* 0.05) in *T. urticae* density among top, middle, and bottom strata within a peppermint plant. In their study, *T. urticae* showed significantly (*p <* 0.05) different preference according to leaf age. Similar to results of Opit et al. [9], more *T. urticae* were found on young-fully-opened (moderately old) leaves than on young and old leaves, although the difference was not significant (*p >* 0.05) between moderately old and old leaves [10]. When considering these statistical results, these studies were also consistent with the results of So [5]. However, they showed conflicting results about vertical distribution of *T. urticae* densities within a plant. Such differences could be related to sampling timing (or *T. urticae* density), sampling methods, and/or host plant. Similar to observation of So [5], the spatial aggregation within a plant could be weak when *T. urticae* densities were increased. The present study focused on the timing of decision making of acaricides’ application for *T. urticae.* Moreover, this study observed all habitable areas compared to previous studies [5,9,10] to take a few samples within a plant. Finally, the host plant in this study was different with those of previous studies [5,9,10].

Although there were significant (*p <* 0.05) differences in *T. urticae* densities within a plant regardless of its developmental stage, the within-plant distribution was slightly different according to its developmental stage. Densities of *T. urticae* adults and immatures were the highest on leaves at the sixth and eighth stems within a strawberry plant according to farms. However, its eggs were found more on slightly younger leaves than its motiles. This result indicated that motiles of *T. urticae* might move to younger leaves to increase the survivorship of their progenies. This phenomenon is well-known in whiteflies [20]. However, its upward movement was unclear compared to whiteflies. For numbers of *T, urticae*, they were different, although the difference was not statistically significant (*p >* 0.05). Moreover, the vertical difference in peak leaves between eggs and motiles was just one or two strata.

Although sampling plans for *T. urticae* have been suggested in multiple studies [8,9,10,11,12], the sampling unit has been suggested only in limited studies [9,12]. Choe et al. [12] have reported that one leaflet as a sampling unit is better than two or three leaflets among strawberries’ trifoliated leaves when considering efficiency. However, they did not discuss which stratum should be sampled. Opit et al. [9] have suggested that a young-fully-opened leaf is the optimal sampling unit in ivy geranium because the density of *T. urticae* is higher on these leaves than on young or old leaves. Based on CV and *r*^2^ values of the present study, young-fully-opened leaflets were also selected as optimal sampling units of *T. urticae* in strawberries. More specifically, the trifoliate leaf in the sixth oldest stem was the best sampling unit of *T. urticae* eggs in strawberries. For its motiles, it would be better to select younger leaves than those of the sixth oldest stem. This suggestion is based on this study’s observation that variation of *T. urticae* increased more than the increase of its density on leaves of more than sixth oldest stems among young-fully-opened leaves. Although there were differences in the trifoliate leaf with high density, low CV and high *r*^2^ values between the two farms, the trifoliate leaf on the fifth oldest stem would be the best sampling unit of *T. urticae* motiles within a strawberry. When both *T. urticae* eggs and motiles were used for sampling, both trifoliate leaves of the fifth or sixth oldest stems were the best sampling unit within a strawberry.

This study suggested the required sample number as a density-dependent way. For research purposes, it is highly recommended to decide sample numbers within a strawberry by counting *T. urticae* numbers on a few leaflets of young-fully-opened leaves. However, the sample number for *T. urticae* within a strawberry could be suggested because its action threshold, ten motiles per leaflet, has already been mentioned in a previous study [12]. To meet precision (*D*) level of 0.3, the required sample number of *T. urticae* motiles was 1.1, indicating at least two leaflets. However, the sample number per plant might be just one leaflet for *T. urticae* egg at *D* = 0.3 based on the proportion of eggs and motiles in this study.

## Figures and Tables

**Table 1:** Observed plant number, average density, coefficient of variation (CV), and coefficient of determination (*r*^2^) of *T. urticae* eggs according to leaf age within a strawberry plant.

Leaf Age ^1^	Observed Number	Average Density ^2^ (Mean ± SE)	CV ^3^	*r* ^2,4^
1	65	11.4 ± 6.23 c ^5^	4.44	0.09
2	65	10.5 ± 2.67 c	2.04	0.66
3	65	33.1 ± 10.31 bc	2.51	0.24
4	65	69.5 ± 20.08 abc	2.33	0.57
5	65	91.5 ± 20.65 ab	1.82	0.52
6	64	139.0 ± 30.32 a	1.74	0.71
7	59	71.2 ± 17.09 abc	1.84	0.55
8	45	85.5 ± 22.28 abc	1.75	0.27
9	20	111.1 ± 38.24 abc	1.54	0.53
10	8	34.6 ± 20.34 abc	1.66	0.60
11	4	28.5 ± 22.62 abc	1.59	0.41
12	1	69	- ^6^	-
Flower cluster	65	13.9 ± 3.42 c	1.98	0.17

^1^ Lower number indicates younger leaf. ^2^ Average number of *T. urticae* per a trifoliate leaf (three leaflets). ^3^ CV was calculated by dividing mean with standard deviation. ^4^ *r*^2^ was calculated from the linear relationship between *T. urticae* density on a selected leaf or whole leaves within a plant. ^5^ Means within a column followed by the same letter are not significantly different (*p >* 0.05; Tukey’s HSD test). ^6^ Calculation is impossible due to limited data number.

**Table 2:** Observed plant number, average density, coefficient of variation (CV), and coefficient of determination (*r*^2^) of *T. urticae* motiles according to leaf age within a strawberry plant in the first farm.

Leaf Age ^1^	Observed Number	Average Density ^2^ (Mean ± SE)	CV ^3^	*r* ^2,4^
1	32	3.7 ± 1.56 b ^5^	2.42	0.54
2	32	10.9 ± 4.06 b	2.10	0.73
3	32	22.8 ± 10.52 ab	2.61	0.23
4	32	35.7 ± 15.12 ab	2.39	0.76
5	32	92.4 ± 26.64 ab	1.63	0.66
6	31	83.1 ± 28.96 ab	1.94	0.40
7	29	88.9 ± 35.07ab	2.12	0.69
8	25	111.9 ± 36.54 a	1.63	0.37
9	11	93.2 ± 40.63 ab	1.45	0.69
10	4	24.3 ± 17.89 ab	1.48	0.82
11	1	118	- ^6^	-
Flower cluster	32	10.3 ± 5.78 b	3.17	0.42

^1^ Lower number indicates younger leaf. ^2^ Average number of *T. urticae* per a trifoliate leaf (three leaflets). ^3^ CV was calculated by dividing mean with standard deviation. ^4^ *r*^2^ was calculated from the linear relationship between *T. urticae* densities on a selected leaf and whole leaves within a plant. ^5^ Means within a column followed by the same letter are not significantly different (*p >* 0.05; Tukey’s HSD test). ^6^ Calculation is impossible due to limited data number.

**Table 3:** Observed plant number, average density, coefficient of variation (CV), and coefficient of determination (*r*^2^) of *T. urticae* motiles according to leaf age within a strawberry plant in the second farm.

Leaf Age ^1^	Observed Number	Average Density ^2^ (Mean ± SE)	CV ^3^	*r* ^2,4^
1	33	1.7 ± 1.08 b ^5^	3.71	0.05
2	33	2.4 ± 1.23 b	2.92	0.40
3	33	7.1 ± 2.48 ab	2.02	0.44
4	33	21.1 ± 7.54 ab	2.06	0.68
5	33	25.6 ± 9.83 ab	2.21	0.36
6	33	51.2 ± 19.96 a	2.24	0.58
7	30	24.1 ± 12.09 ab	2.75	0.32
8	20	37.4 ± 24.34 ab	2.91	0.20
9	9	24.2 ± 15.25 ab	1.89	0.24
10	4	1.8 ± 0.85 ab	0.98	0.01
11	3	1.7 ± 1.67 ab	1.73	0.90
12	1	3	- ^6^	-
Flower cluster	33	6.7 ± 2.52 ab	2.17	0.02

^1^ Lower number indicates younger leaf. ^2^ Average number of *T. urticae* per a trifoliate leaf (three leaflets). ^3^ CV was calculated by dividing mean with standard deviation. ^4^ *r*^2^ was calculated from the linear relationship between *T. urticae* densities on a selected leaf and whole leaves within a plant. ^5^ Means within a column followed by the same letter are not significantly different (*p >* 0.05; Tukey’s HSD test). ^6^ Calculation is impossible due to limited data number.

**Table 4:** Observed plant number, average density, coefficient of variation (CV), and coefficient of determination (*r*^2^) of *T. urticae* (both eggs and motiles) according to leaf age within a strawberry plant.

Leaf Age ^1^	Observed Number	Average Density ^2^ (Mean ± SE)	CV ^3^	*r* ^2,4^
1	65	14.1 ± 6.97 c ^5^	3.99	0.09
2	65	17.2 ± 4.41 c	2.07	0.71
3	65	47.9 ± 15.4 bc	2.59	0.25
4	65	97.8 ± 25.95 abc	2.14	0.59
5	65	149.9 ± 33.22 ab	1.79	0.60
6	64	205.6 ± 46.21 a	1.80	0.61
7	59	127.1 ± 34.15 abc	2.06	0.61
8	45	164.3 ± 44.36 ab	1.81	0.31
9	20	173.3 ± 61.92 abc	1.60	0.61
10	8	47.6 ± 29.30 abc	1.74	0.65
11	4	59.3 ± 51.56 abc	1.74	0.46
12	1	72	- ^6^	-
Flower cluster	65	22.39 ± 5.90 c	2.13	0.23

^1^ Lower number indicates younger leaf. ^2^ Average number of *T. urticae* per a trifoliate leaf (three leaflets). ^3^ CV was calculated by dividing mean with standard deviation. ^4^ *r*^2^ was calculated from the linear relationship between *T. urticae* densities on a selected leaf and whole leaves within a plant. ^5^ Means within a column followed by the same letter are not significantly different (*p >* 0.05; Tukey’s HSD test). ^6^ Calculation is impossible due to limited data number.

**Table 5:** Densities (mean ± SE) of *T. urticae* according to relative locations of leaflets among a trifoliate leaf (density was calculated by summing *T. urticae* numbers on all leaflets at a location, e.g., left, middle, and right, per plant).

Developmental Stage	Site	Left	Middle	Right
Egg	Both farms	153.6 ± 49.15 a ^1^	201.1 ± 65.62 a	163.2 ± 53.73 a
Motile	First farm	123.3 ± 57.01 a	177.8 ± 81.97 a	151.5 ± 75.65 a
Motile	Second farm	49.2 ± 23.84 a	68.1 ± 33.95 a	43.3 ± 18.11 a
Both (Egg + Motile)	Both farms	239.3 ± 83.11 a	323.2 ± 111.49 a	259.8 ± 94.53 a

^1^ Means within a row followed by the same letter are not significantly different (*p >* 0.05; Tukey’s HSD test).

## Data Availability

Data used in this study are available from the corresponding authors upon reasonable request.
